# Comparison of *Auxenochlorella protothecoides* and *Chlorella* spp. Chloroplast Genomes: Evidence for Endosymbiosis and Horizontal Virus-like Gene Transfer

**DOI:** 10.3390/life12030458

**Published:** 2022-03-20

**Authors:** Sang-Hyuck Park, John A. Kyndt, Judith K. Brown

**Affiliations:** 1 School of Plant Sciences, The University of Arizona, Tucson, AZ 85721, USA; sanghyuck.park@csupueblo.edu (S.-H.P.); jbrown@ag.arizona.edu (J.K.B.); 2 Institute of Cannabis Research, Colorado State University-Pueblo, Pueblo, CO 81001, USA; 3 College of Science and Technology, Bellevue University, Bellevue, NE 68005, USA

**Keywords:** *Auxenochlorella protothecoides* UTEX 25, biofuel production, chloroplast genome, horizontal gene transfer

## Abstract

Resequencing of the chloroplast genome (cpDNA) of *Auxenochlorella protothecoides* UTEX 25 was completed (GenBank Accession no. KC631634.1), revealing a genome size of 84,576 base pairs and 30.8% GC content, consistent with features reported for the previously sequenced *A. protothecoides* 0710, (GenBank Accession no. KC843975). The *A. protothecoides* UTEX 25 cpDNA encoded 78 predicted open reading frames, 32 tRNAs, and 4 rRNAs, making it smaller and more compact than the cpDNA genome of *C. variabilis* (124,579 bp) and *C. vulgaris* (150,613 bp). By comparison, the compact genome size of *A. protothecoides* was attributable primarily to a lower intergenic sequence content. The cpDNA coding regions of all known *Chlorella* species were found to be organized in conserved colinear blocks, with some rearrangements. The *Auxenochlorella* and *Chlorella* species genome structure and composition were similar, and of particular interest were genes influencing photosynthetic efficiency, i.e., chlorophyll synthesis and photosystem subunit I and II genes, consistent with other biofuel species of interest. Phylogenetic analysis revealed that *Prototheca cutis* is the closest known *A. protothecoides* relative, followed by members of the genus *Chlorella*. The cpDNA of *A. protothecoides* encodes 37 genes that are highly homologous to representative cyanobacteria species, including *rrn16*, *rrn23*, and *psbA*, corroborating a well-recognized symbiosis. Several putative coding regions were identified that shared high nucleotide sequence identity with virus-like sequences, suggestive of horizontal gene transfer. Despite these predictions, no corresponding transcripts were obtained by RT-PCR amplification, indicating they are unlikely to be expressed in the extant lineage.

## 1. Introduction

Species in the *Auxenochlorella* and *Chlorella* genera (phylum Chlorophyta) are eukaryotic, single-celled, spherically shaped photosynthetic green algae of about 2–10 μm in size. They reside primarily in fresh water and terrestrial habitats, and some species are adapted to brackish and marine environments. A number of Chlorophyta species are of commercial interest for use as food supplements [1]; for cosmetics [2], dietary [3], and pharmaceutical products [4]; and in the production of specialized chemicals [5]. In particular, several Chlorophyta species have been exploited as renewable feedstocks for biofuel production because of their capacity to metabolize carbon from CO_2_ and produce large amounts of carbon stored as biomass, owing to the ability of cells to divide at least four times within 20–24 h [6]. To advance the industrial uses of green microalgae, recent research goals have centered on better understanding the effects of variable CO_2_ concentrations, nutrients, and light sources [7,8,9]. Other studies have emphasized algal cultivation approaches in different production platforms [10], as well as improving genotype characteristics and traits through genetic engineering [11] and laboratory-adaptive evolution [12].

One species of great interest for biofuel production is *Auxenochlorella protothecoides* (Krűger) Kalina et Punčochářová (previously *C. protothecoides*), a freshwater mixotrophic microalga belonging to the *Chlorellaceae* family. A recent phylogenetic analysis of the 5.8S rRNA and (nuclear) ITS-2 sequence has placed *A. protothecoides* in the Scotielloideae subfamily, with *Chlorella* species relatives *C. heliozoae, C. lobophora, C. sorokiniana*, *C. variabilis,* and *C. vulgaris,* subfamily Chlorelloideae (*Chlorellaceae*) [13].

Taxonomically, Chlorophyta comprises six diverse classes, including the chlorophyte core and the paraphyletic prasinophytes. The core families consist of Chlorophyceae, Chlorodendrophyceae, Pedinophyceae, Trebouxiophyceae, and Ulvophyceae, which are morphologically diverse and characterized by distinct modes of cell division [14]. In contrast, the prasinophytes, which diverged earlier than the chlorophytes, vary morphologically with respect to cell shape, size, flagella type, and mitotic and cytokinetic mechanisms [14]. A recent phylogenomic analysis showed that Chlorophyceae is a monophyletic group, whereas Ulvophyceae, Trebouxiophyceae, and Prasinophyceae are polyphyletic [15]. 

Chloroplast genomes (cpDNA) are relatively small and encode certain highly conserved genes, making it possible to identify informative gene sequences and/or to use the complete genome sequence, when available, for taxonomic studies [16]. Non-coding sequences are also major components of the algal chloroplast genome, which includes non-repeated intergenic sequences (i.e., algal introns), repeated sequences, and transposable elements (TE). It has been reported that introns populated by repeated sequences account for 78% of the genome size in the green alga *Floydiella terrestris* [17]. Repeated sequences and TEs are the main resources for the events of gene duplication, deletion, and gene rearrangements [18], which contribute to genome diversification and have an impact on genome evolution [19]. Therefore, identifying non-coding sequences and comparing the identified sequences across related species in the same lineages will provide a clue about the diversification process of the species. 

Algal chloroplasts evolved from an endosymbiotic relationship with cyanobacteria, with which they share some similar genome structure and predicted gene functions [20]. Comparative analyses of green algal chloroplast and cyanobacterial genome sequences revealed that the quadripartite structure and gene partitioning patterns characteristically found in modern green algae are cyanobacterially derived [21]. 

There is ample evidence for horizontal gene transfer (HGT) between prokaryotic and eukaryotic genomes. Introgression of foreign genes can influence genetic diversity and fitness [22]. Such virus-like sequences may consist of mobile genetic elements, plasmids, prophages, and phages [23]. Tracing the origins of genes encoded by plastid genomes has led to new insights about genome evolution in the recipient host and clarified early symbiotic interaction(s) between bacteria and green algae [24]. In addition to prokaryotic coding regions, horizontal transfer of virus-like elements has occurred widely between bacterial genomes, as well as to algae from bacterial endosymbionts. 

Approximately 166 green algal chloroplast genome sequences are available in the NCBI Organelle Genome Resources database (https://www.ncbi.nlm.nih.gov/genome/organelle/) (last accessed on 10 February 2022), including 38 Trebouxiophyceae and 16 Chlorellaceae members. The size of chloroplast genomes ranges from 48 kb (*Prototheca stagnorum*) to 1.35 Mb (*Haematococcus lacustris*) [25], with most variation accounted for by non-coding gene content and the number of copies of inverted, i.e., repeated sequences [26]. The quadripartite genome structure found in many green microalgal chloroplast genomes consists of two copies of a large inverted repeat sequence that divides the genome into small and large single-copy regions [24]. In general, nuclear genome size has been found to be correlated with organismal size [27], with a small genome size considered beneficial through the maximization of energy, nutrients, and/or space requirements [28]. 

The availability of algal and higher plant chloroplast genome sequences has enhanced the understanding of their evolutionary relationships by strengthening the reliability of taxonomic classification [29] and facilitating reassessment of previous species classifications [30] to reveal new insights into global genomic and evolutionary relationships. Recent genome analyses have revealed subspecies divergence among *C. sorokiniana* variants [31], also contributing to a better understanding of gene composition, as well as photosynthetic and metabolic pathways conducive to genetic modification [32,33]. 

In this study, the complete chloroplast genome (cpDNA) of *A. protothecoides* isolate UTEX 25 was resequenced and annotated. The cpDNA for three isolates of *A. protothecoides*, *C. variabilis*, and *C. vulgaris* were aligned. The genome structures were compared, with particular attention to genes with a predicted involvement in metabolic pathways, chloroplast biogenesis, photosynthesis, and fatty acid synthesis. The evolutionary relationships of 26 predicted chloroplast proteins were analyzed phylogenetically for 37 algal species belonging to the class Trebouxiophyceae; three terminal taxa in the class Pedinophyceae, the two *A. protothecoides* isolates; and the two outgroups belonging to the class Prasinophytes, *Micromonas pusilla* and *Ostreococcus tauri*. Finally, based on the potential for horizontal gene transfer from ancestral marine cyanobacterial endosymbionts to the three extant microalgal species of interest, cyanobacterial-like and virus-like elements were found to be encoded on the *A. protothecoides* UTEX 25 cpDNA by in silico analysis. To determine whether selected HGT candidates were expressed in vivo, primers were designed and used for RT-PCR amplification of the respective transcripts. 

## 2. Materials and Methods

### 2.1. Auxenochlorella protothecoides UTEX 25 Culture and DNA Isolation 

The *A. protothecoides* isolate UTEX 25 was obtained from the UTEX Culture Collection of Algae (https://utex.org) (last accessed on 10 February 2022). The cultures were established in 500 mL of Bristol medium (2.94 mM NaNO_3_, 0.17 mM CaCl_2_·2H_2_O, 0.3 mM MgSO_4_, 0.43 mM K_2_HPO_4_, 1.29 mM KH_2_PO_4_, and 0.43 mM NaCl) with gentle shaking at 28 °C, with a continuous flow of 5% CO_2_ under 108–135 PPF (μmol/m^2^·s; 8000–10,000 lux) for 14–18 days, until biomass was approximately 300 mg cells/flask by wet weight. Cells were harvested by centrifugation at 4500× *g* at 4 °C for 8 min. The pellet was washed twice in double-distilled water (ddH_2_0), and total DNA was isolated using a QIAGEN plasmid mini kit (QIAGEN Inc., Valencia, CA, USA) according to the manufacturer’s instructions.

### 2.2. Auxenochlorella protothecoides UTEX 25 Chloroplast Genome Sequencing and Annotation

The *A. protothecoides* cpDNA was assembled from Illumina HiSeq 2000 and 454 DNA reads. A 100 base-pair (1 × 100 bp) Illumina shotgun library was prepared from total DNA isolated from the established *A. protothecoides* lab culture according to the manufacturer’s protocol for sequencing on the TruSeq platform. Libraries were sequenced using an Illumina GAII sequencer. The Illumina sequences consisting of ~2 billion reads were assembled with VELVET (version 1.0.13) [34]. Shotgun single-end and paired-end (11 kb insert) DNA libraries were prepared, and the sequence was determined using the 454 Titanium platform. Newbler version 2.3, release 091027_1459, was used to assemble the resultant 1.16 and 1.15 million reads obtained for each replicate, respectively. 

The consensus sequence was determined from VELVET; the Newbler assemblies were shredded into 10-kb fragments and re-assembled with reads from the 454 paired-end library by Phrap (version 1.080812, High-Performance Software, LLC) [35]. The chloroplast sequence was identified within the ‘hybrid assembly’ through a BLASTN search of *C. variabilis* [36] and *C. vulgaris* cpDNA available in the GenBank Database (GB accession nos. NC_015359 and NC_001865, respectively). Potential misassemblies were analyzed and corrected using Dupfinisher [37]. The accuracy of the repeated sequence regions was evaluated by constructing a circular consensus sequence using Consed [38], resulting in >2000× coverage from the combined platforms. The chloroplast genome coding and non-coding regions were annotated using the Dual Organellar GenoMe Annotator (DOGMA) and rapid-annotation subsystem technology (RAST) [39,40]. The genome maps were drawn using SeqBuilder (DNASTAR, Madison, WI, USA). 

### 2.3. Chloroplast Genome Sequence Alignment 

To examine whether large-scale evolutionary events, such as gene rearrangement, loss, duplication, and inversion, were evident between *A. prototheocides*, *C. vulgaris*, and *C. variabilis*, multiple genome alignments were conducted using a software package called Mauve, version 2.3.1 (http://darlinglab.org/mauve/user-guide/introduction.html) (last accessed on 10 February 2022). The Mauve algorithm is designed to align orthologous and horizontally transferred genomic (xenologous) regions that have undergone both local and large-scale changes [41].

### 2.4. Intron and Repeat Element Analysis

To identify algal introns within the chloroplast genomes of interest, the Group I Intron Sequence and Structure Database (GISSD; http://www.rna.whu.edu.cn/gissd/) (last accessed on 10 February 2022) [42] and the mobile group II intron database (http://www.fp.ucalgary.ca/group2introns/) (last accessed on 23 May 2014) [43] were used to identify group I and group II introns, respectively. RepeatMasker (http://www.repeatmasker.org) (last accessed on 10 February 2022) was used to detect interspersed repeated sequences and transposable-element (TE)-like repeats. Briefly, the cp genome sequence of *A. protothecoides* UTEX 25 was aligned to the repeat library of diatom *Thalassiosira pseudonana*, which is available in the RepeatMasker database (https://www.girinst.org/repbase/) (last accessed on 10 February 2022), and cross_match was used to identify the repeat elements [44]. 

### 2.5. Bacterial and Viral Sequence Search

To search for potential bacterial and prophage sequences in the cpDNA of *A. protothecoides* UTEX 25, the NCBI BLASTN tool (https://blast.ncbi.nlm.nih.gov/Blast.cgi?PAGE_TYPE=BlastSearch) (last accessed on 10 February 2022) was used to search the cyanobacteria (taxid:1117) and virus (taxid:10239) libraries, respectively.

### 2.6. Phylogenetic Analyses

The gene phylogeny of the concatenated chloroplast was estimated using a Bayesian Markov chain Monte Carlo method and maximum likelihood (ML) algorithms, implemented in MrBayes v3.2.6 [45] and RAxML version 8.2.10 [46]. The sequence matrix contained 26 amino acid sequences for algal proteins (Supplementary Table S2) selected from 37 species belonging to the class Trebouxiophyceae, including two isolates of *A. protothecoides* and three terminal taxa in the class Pedinophyceae. The prasinophyte algae *Micromonas pusilla* and *Ostreococcus tauri* were used as the outgroups. The selection of genes for the analysis was based on those previously reported to be informative for estimating chloroplast phylogenies in Trebouxiophyceae [15,31]. Multilocus species trees were not reconstructed in this study because all chloroplast genes effectively belong to the same locus. Therefore, they are expected to be less influenced by incomplete lineage sorting than nuclear genes due to the reduced effective population sizes of chloroplast genomes [47], making them more likely to accurately reconstruct the “species tree” than nuclear genes. 

Complete gene sequences were unavailable for certain taxa included in the analysis, amounting to 1.37% of cells in the data matrix and resulting in gaps comprising 14.75% of the alignment. Amino acids for each gene were aligned using Muscle v3.8.31 [48]. The best-fitting combination of partitioning scheme and substitution models was determined using PartitionFinder version 2.1.1 [49] with Akaike information criterion (AIC) and branch lengths linked across partitions. Twenty-six possible partitions were initially defined (one per protein), and the best-fitting strategy had 17 data blocks (Supplementary Table S2). Four independent Bayesian runs of four chains each (three heated and one cold chain) were carried out for 5 × 10^6^ generations, with a burn-in of 1 × 10^6^ generations. Trees were sampled every 100 generations. The analyses were considered to have adequately sampled the solution space based on the standard deviation cutoff of split frequencies, which was below 5 × 10^−3^.

### 2.7. Analysis of Four Predicted Virus-like Transcripts 

The blast search of the virus library with the cpDNA of *A. protothecoides* revealed hits to four predicted virus-like sequences: Stealth_rrn16, Stealth_rrn23, Prochlorococcus_psbA, and cyanophage_psbA. To verify the presence or absence bioinformatically identified virus transcripts, primers were designed and used in reverse transcriptase PCR (RT-PCR) amplification reactions with cDNA synthesized from *A. protothecoides* total RNA. Algal total RNA was isolated using the RNeasy plant mini kit (Qiagen, Valencia, CA, USA). First-strand cDNA synthesis was carried out using SuperScript^TM^ III reverse transcriptase (Invitrogen, Grand Island, NY, USA). The reaction contained 1 µL of 50 µM oligo (dT)_20_, 1 µL of 10 mM dNTPs, and 1 µg total RNA in 20 µL. The reaction was incubated at 65 °C for 5 min and held on ice for 1 min, and reverse transcription was carried out using SuperScript^TM^ III RT according to the manufacturer’s protocol. The *A. protothecoides* UTEX 25 cpDNA was used as template to RT-PCR amplify active transcripts of the two predicted virus-like sequences. The RT-PCR amplification reactions were carried out using the following primers, designed around the 18S rDNA and two virus-like *psbA* and *rrn23* sequences each, Apro_F18S: 5′-GGGTTCGATTCCGGAGAG-3′and Apro_R18S: 5′-GTACAAAGGGCAGGGACGTAAT-3′ (1.5 kb; as the positive control), Apro_PsbA_F: 5′-TTACCCAATCTGGGAAGCTG-3′ and Apro_PsbA_R: 5′-ATACCAACAACTGGCCAAGC-3′ (528 bp), Prochlorococcus_PsbA_F: 5′-ATGTCTTCTAGCTGCAACAACATGC-3′ and Prochlorococcus_PsbA_R: 5′-TAGTTCTGTGAATCTAAACCAGTG-3′ (588 bp), Stealth_rrn16_F: 5′-TGGTGACAGTGGGCAGCA-3′ and Stealth_rrn16_R: 5′-GACCCTACCGTGGTCACCTG-3′ (230 bp), and Stealth_rrn23_F 5′-CGCTTGAGAGAACTGCGTTGA-3′ and Stealth_rrn23_R: 5′-GTTGGAGACAGCGGGGAAG-3′ (331 bp). Amplicons were cloned into the pGEM-T Easy plasmid vector (Promega Inc, Madison, WI, USA) according to the manufacturer’s instructions and sequenced bidirectionally by capillary Sanger sequencing at the University of Arizona Genetics Core (https://uagc.arl.arizona.edu/node/32) (last accessed on 10 February 2022).

## 3. Results

### 3.1. General Characteristics

**Figure 1:**
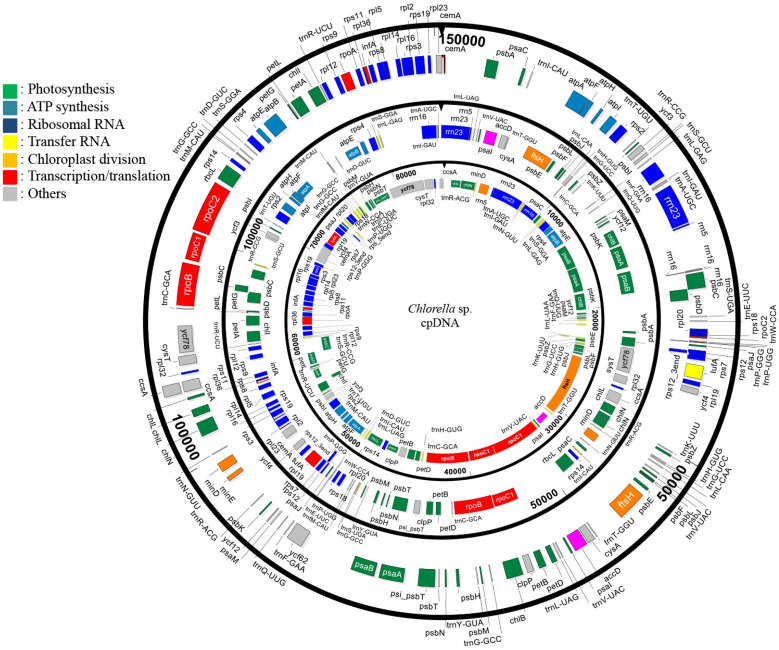
The chloroplast genomes of *Auxenochlorella protothecoides* (84.5 kb; inner circle), *Chlorella variabilis* (124.5 kb, middle circle), and *Chlorella vulgaris* (150 kb; outer circle). Genes are color-coded based on their conserved metabolic function (see legend for categories).

The *A. protothecoides* cpDNA was assembled de novo from Illumina and 454 reads into a complete, circular molecule of 84,579 bp (Figure 1). The complete sequences of the *A. protothecoides* cpDNA were deposited in the NCBI GenBank Database, (accession no. KC631634.1). The *A. protothecoides* cpDNA genome had 30.8% GC content and encoded 32 tRNAs, 4 rRNAs, and 78 protein-encoding genes. The UTEX 25 cpDNA sequence shared 99.9% nucleotide identity with the cpDNA sequence of *A. protothecoides* UTEX 2341 (GenBank accession no. KC843975) [50], varying by one single-nucleotide polymorphism (SNP) and four insertions/deletions (indels). Two additional tRNAs and one additional rRNA sequence were identified, a discrepancy possibly due to the annotation software used to annotate the cpDNA sequences. The *A. protothecoides* chloroplast genome was 32% and 44% smaller in size than the chloroplast genome of *C. variabilis* and *C. vulgaris*, at 124,579 and 150,613 bp, respectively. Despite the compact size of the genome, the number of genes encoded by the *A. protothecoides* chloroplast genome was similar to that of *C. variabilis* and *C. vulgaris,* at 113 and 120 open reading frames (ORFs), respectively. The small size of *A. protothecoides* was attributable to fewer non-coding sequences (19%), at 46% and 53% in the *C. variabilis* and *C. vulgaris* chloroplast genomes, respectively. 

Genomic variation was evaluated by conducting a global analysis of the coding regions (Supplementary Table S1). Approximately 95% (n = 105, excluding paralogs and duplications) of the genes identified in *A. protothecoides* cpDNA were conserved in the two other Chlorella chloroplast genomes. In contrast, several ‘unique’ genes were encoded by *A. protothecoides*, including a transfer RNA (trnL-UAA), a ribosomal protein small subunit (rps12_5), a tRNA (Ile)-lysidine synthetase (Tils), and two predicted genes of unknown function. With respect to paralogs, a gene encoding protoporphyrin IX Mg-chelatase subunit (ChlI) was uniquely duplicated in *A. protothecoides*, whereas the photosystem II subunit (PsbA and PsbC), tRNA (trnC-GCA, trnG-GCC), and light-independent protochlorophyllide oxidoreductase (*chlN*) genes were only duplicated in *C. variabilis*. Two genes encoding a light-independent protochlorophyllide reductase iron-sulfur ATP-binding protein (ChlL) and the RNA polymerase β′′ subunit (RpoC2) were duplicated only in *C. vulgaris.* Within the putative ‘gene insertions/deletions’ (indels), the *A. protothecoides* chloroplast genome lacked genes for the predicted cell-cycle gene, *ycf62*, and the cell-division-related gene, *minE*. Despite the extensive conservation observed among most genes identified for all three of the Chlorellaceae chloroplast genomes, each species showed unique attributes based on gene order and architecture of the chloroplast genome (Supplementary Figure S1). Functionally related predicted genes involved in ATP synthesis, photosynthesis, and transcription, as well as the ribosomal RNAs genes, were identified in all three species; however, each algal species also showed distinctive syntenies and/or harbored unique gene arrangements (Figure 1). 

### 3.2. Phylogenetic Analysis of Trebouxiophyceae Chloroplast Genomes 

**Figure 2:**
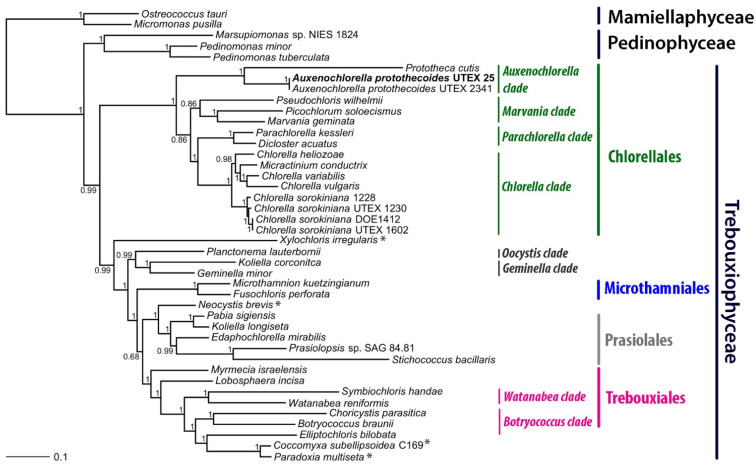
Phylogenetic analysis of Trebouxiophyceae chloroplast genomes based on 26 concatenated chloroplast proteins (Supplementary Table S2) using Bayesian approaches. Numbers indicate Bayesian posterior probabilities. Representatives from the related Pedinophyceae and Mamiellaphyceae classes were included in the comparison. * Trebouxiophyceae *ordo incertae sedis*.

The chloroplast phylogenetic tree of 37 algal species in the class Trebouxiophyceae was constructed using Bayesian (Figure 2) and maximum likelihood (Supplementary Figure S2) methods. Overall, the phylogenetic topology is consistent with that reported in previous analyses; however, the topology within Chlorellales is somewhat different [50]. In the previous analysis, only 6 species of Chlorellales were included, as opposed to 16 in our study, which could explain the smaller topological rearrangements. Nevertheless, Chlorellales contain the same Chlorella, Marvania, and Parachlorella clades as observed before [50]; however, this is now expanded with a clearly distinct Auxenochlorella clade. The trees concordantly place two *A. protothecoides* strains, UTEX 25 and 2341, as sister to *Prototheca cutis*, consistent with previous phylogenetic analyses [51]. The strain UTEX 2341 was previously known as *C. minutissima* but has been reclassified as *A. protothecoides* [52]. The main phylogenetic clades within the core Threbouxiophyceae are observed as in [50], with the separated Microthamniales and Prasiolales clades, the Oocystis and Geminella clades closely related, and the Watanabea and Botryococcus clades as part of the Trebouxiales order. Several species in the Trebouxiophyceae class are *ordo incertae sedis*, and further investigation will undoubtedly expand these clades. As seen before, the Chlorellales order forms a clearly distinct clade, separated from what was described as the ‘core Trebouxiophyceae’ [50]. Similarly to Figure 2, previous analyses predicted Chlorellales as a sister group to Pedinophyceae [50]. Our analyses do not reconstruct this latter relationship as closely (Figure 2 and Supplementary Figure S2); however, our analyses do not conflict with previous phylogenetic estimates [50] and are consistent with consensus relationships within Trebouxiophyceae [14].

### 3.3. Plastid-Encoded RNA Polymerase

The plastid-encoded RNA polymerase is a multi-protein complex composed of RpoA, RpoB, RpoC1, and RpoC2 subunits, which are homologous to those of *Escherichia coli*, α, β, β′, and β′′ [53]. These core subunits, RpoA, RpoB, RpoC1, and RpoC2, are identified and annotated in *A. protothecoides*, *C. variabilis,* and *C. vulgaris* chloroplast genomes. 

### 3.4. Chloroplast Division

Analogous to *E. coli* cell division, the plastid cell division of *C. vulgaris* is known to initiate with the formation of a macromolecular machine called a divisome [54]. The divisome is formed by polymerization of the tubulin-like protein FtsZ into a ring-like structure at a mid-cell site [55]. The ring-like structure, or ‘Z-ring’, gives rise to plastid division [56]. During cytokinesis, placement of the Z-ring site at the mid-cell plane results in rapid oscillation of the multiprotein complex MinC and MinD between each pole, thereby preventing Z-ring formation from becoming displaced from the mid-cell plane. After initiation of cytokinesis, the MinE protein inhibits the MinCD complex, thereby allowing Z-ring formation to occur [57]. The predicted antagonistic interaction affecting the MinCD complex in both *A. protothecoides* and *C. variabilis* is expected to be mediated by the zinc-metalloprotease FtsH protein, which degrades the bacterial cell-division protein FtsZ in vitro [58]. However, in *C. vulgaris*, this predicted interaction appears to be mediated by both MinE and FtsH, or at least both are present. These observations have led to the following hypotheses: (1) *A. protothecoides* employs a cell-division mechanism similar to that of *C. variabilis* based on the presence of *MinD* and *FtsH*; and, in contrast, (2) the chloroplast-division mechanism of *C. vulgaris* is mediated by both *MinE* and *FtsH*, suggesting the differential evolutionary favorability of distinct mechanisms among these algal species, despite being close relatives. 

### 3.5. Chlorophyll Synthesis

**Figure 3:**
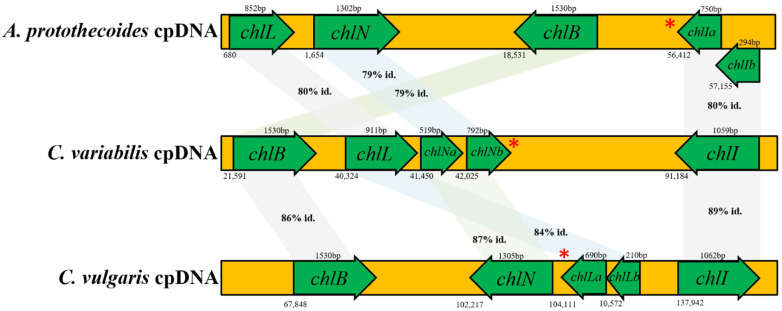
Genomic arrangements of chlorophyll synthesis genes. The percentages indicate the nucleotide sequence identities between two algal species. The similarity was calculated based on sequence similarity scores and % coverage using the National Center for Biotechnology Information (NCBI) BLASTN suite. The asterisk indicates a segmented gene. The number under each green arrow indicates the location of each gene in the chloroplast genome (not drawn to scale). Identity is abbreviated as ‘id.’.

The first step of light-dependent chlorophyll biosynthesis is the ATP-dependent insertion of an Mg^2+^ ion into protoporphyrin, catalyzed by ChlI, ChlD, and ChlH [59]. The ChlD interacts with ChlI to form the ChlI-ChlD complex that then binds Mg^2+^ATP. The magnesium ion that is released from the ChlI-ChlD-Mg^2+^ATP complex is inserted into the ChlH-protoporphyrin IX complex [60]. Based on the results of this study, the *A. protothecoides* chloroplast genome only encoded the *chlI* but not the *chlD* and *chlH*. The *chlI* encoded by *A. protothecoides* showed a high nucleotide sequence similarity (>80%) with the *C. variabilis* and *C. vulgaris* predicted homologs. Despite their shared nucleotide similarity, the two genomic arrangements were found to be quite different (Figure 1 and Supplementary Figure S1). For example, the *A. protothecoides chlI* was separated into two coding regions, *chlIa* (744 bp) and *chlIb* (333 bp), that share 48.2% nucleotide identity (Figure 3), whereas *C. variabilis* and *C. vulgaris* encoded only one ORF. 

Comparative analysis of the genome sequences of the Chlorellaceae exemplars indicated the presence of *chlD* in the *A. protothecoides* nuclear genome. In contrast, *chlH* was not identified (annotated) in the *A. protothecoides* nuclear genome (GenBank accession no. APJO00000000), a result that was possibly due to an annotation error and/or misassembly. In contrast, *chlD* and *chlH* were both annotated in the *C. variabilis* or *C. vulgaris* nuclear genome, respectively, albeit as hypothetical proteins [61]. In a second kind of analysis, tblastx was used to search for the three protein-coding regions in the whole-genome shotgun reads of *C. vulgaris, C. variabilis,* and *A. protothecoides*, revealing three regions of shared homology between *chlD* and *chlI* in the genomes of *C. vulgaris* and *C. variabilis*. However, these complete proteins were not encoded by these regions, suggesting that either the functional counterparts are not present in the assemblies or that the genes have diverged to the extent that they were not identifiable based on amino acid similarity. The genes involved in chlorophyll biosynthesis for the three algal chloroplast genomes were similar but not identical (Figure 3). These observations point to some extent of genome divergence among the three species that may potentially be found to be directly or indirectly associated with extant niche specialization.

Relatively slowly evolving genes are often preferred for phylogenetic analyses, and in Plantae, photosynthetic genes have been shown to be informative of taxonomic relationships [62]. The reduction of protochlorophyllide to chlorophyllide is an essential step in light-‘independent’ chlorophyll biosynthesis [63], a reaction that is catalyzed by a multicomplex protein encoded by three chloroplast genes, *chlB*, *chlL*, and *chlN* [64]. All three of the *Chlorellaceae* spp. studied here contain these three genes, thereby facilitating chlorophyll synthesis, independent of light-mediated synthesis. Notably, whereas *chlN* and *chlL* are single-exon genes in most Chlorellaceae, the *chlN* in *C. variabilis* was found to be encoded as two exons (*chlNa* and *chlNb)* with a 56 bp intron, and the *chlL* in *C. vulgaris* was fragmented into *chlLa* and *chlLb* with a 951 bp intron. The light-independent chloroplast genes, i.e., *chlB*, *chlL*, and *chlN*, in *A. protothecoides* share 77–80% nucleotide sequence identity with their homologs in *C. variabilis* and *C. vulgaris*, whereas in *C. variabilis* and *C. vulgaris*, the putative homologs are 84–87% identical (Figure 3). 

### 3.6. Chloroplast Introns

**Figure 4:**
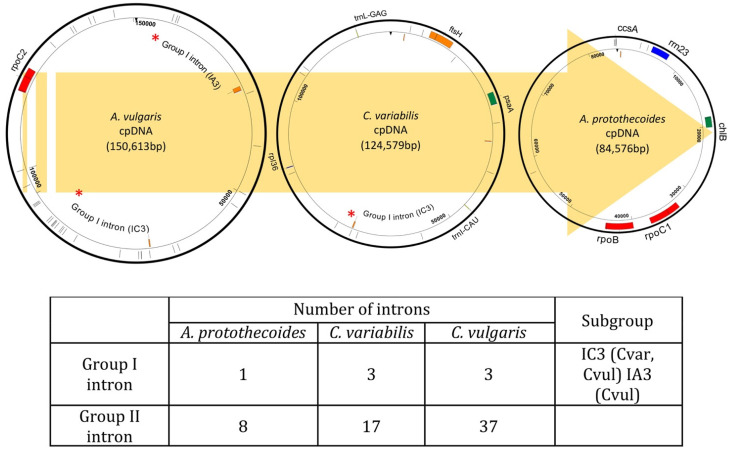
Chloroplast genome sequence map showing group I (orange boxes) and group II introns (green boxes) for the three algal species to which the chloroplast genomes were compared in this study. The yellow arrow indicates the predicted direction of chloroplast evolution. * indicates the position of the introns on the genome. The tabular portion indicates the number and type of intron identified for the three *Chlorella* species.

The *A. protothecoides* chloroplast genome was devoid of non-coding intron sequences between photosynthetic genes, whereas *C. variabilis* and *C. vulgaris* both contained a 56 bp and 951 bp intron located between the two exons of *chlN* and *chlL*, respectively. The *A. protothecoides* cpDNA was found to harbor putative group I and II introns (Figure 4), which are identified in the nuclear, chloroplast, and mitochondrial genomes of a broad range of organisms [65]. These types of introns consist of a catalytic RNA, e.g., a ribozyme, an enzyme known to be involved in RNA splicing, viral replication, and in the biosynthesis of transfer RNAs [42,66]. Most group I introns (>95%) have been found in the chloroplast *tRNA-leu* and belong to the IC3 subgroup [42]. In *A. protothecoides* cpDNA, one group I intron was identified within non-coding sequences, whereas both *C. variabilis* and *C. vulgaris* genomes harbored three group I introns within the non-coding sequences and the genes *rrn23* and *trnL-UUA*. With respect to group II introns, all three species contained only partial fragments (16–65 bp) based on BLASTN analysis using the Archaea *Methanosarcina barkeri* strain Fusaro Group II intron database. 

### 3.7. Transposable Element (TE) and Repeated Sequence Analysis

Table 1 shows the number, length (bp), and percentage of small RNA, simple repeat sequences, and low-complexity repeats present in *A. protothecoides, C. variabilis,* and *C. vulgaris*. Analysis of the repeated sequences revealed that neither the TE elements, DNA transposons, and retroelements nor satellite DNAs were readily apparent among the three *Chlorellaceae* spp. However, small RNAs occurred at 2.23% in *A. protothecoides*, 1.23% in *C. variabilis,* and 0.62% in *C. vulgaris*. In addition, simple DNA repeats (e.g., microsatellites), such as poly-purine/poly-pyrimidine content or regions of high AT or GC content, were found to comprise less than 1% of the cpDNA in all three algal species (Table 1). Notably, *A. protothecoides* had the greatest amount of ‘low-complexity DNA’ (11.9%) in the cpDNA, whereas *C. variabilis* had the least, at 1.49%.

### 3.8. Endosymbiotic Cyanobacteria and Viral Signatures

**Figure 5:**
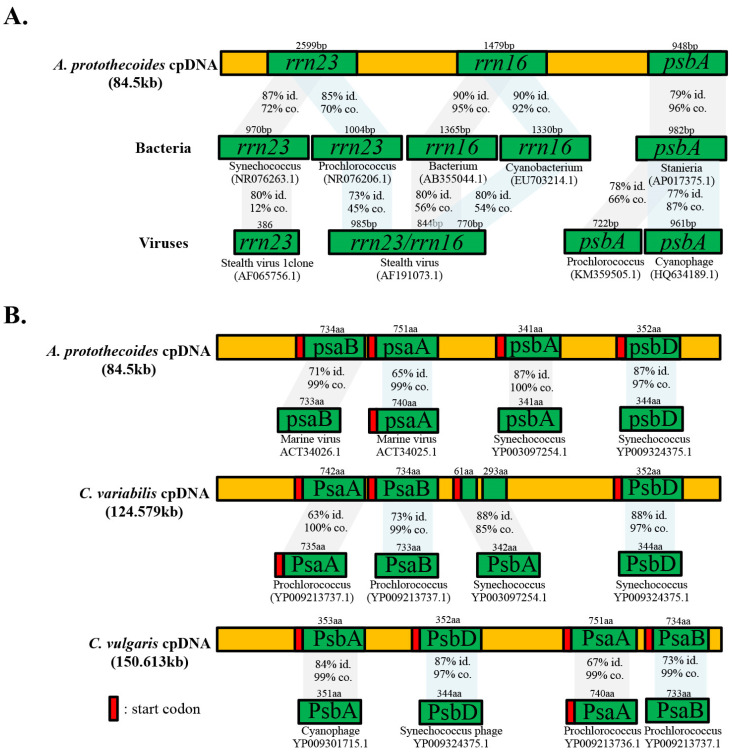
Endosymbiotic relationship of Chlorellaceae with cyanobacteria. (**A**) Schematic drawing representing gene nucleic acid homologs between *Auxenochlorella protothecoides* cpDNA and marine bacteria, as well as between marine bacteria and viruses. (**B**) Schematic drawing representing amino acid homologs between *Chlorellaceae* spp. and marine viruses (not drawn to scale). Identity and coverage are abbreviated as ‘id.’ and ‘co.”, respectively.

To corroborate the evolutionary relationship of the *A. protothecoides* chloroplast genome with its presumed cyanobacterial ancestor(s) the cpDNA sequence was blasted against the NCBI cyanobacterial genome database. At least 37 *A. protothecoides* genes, including *rrn16* and *rrn23*, which were among the top-scoring hits, shared high sequence similarity scores, ranging from 86% to 89%, with cyanobacterial sequences available in the NCBI GenBank database (e-value: 0 and query coverage >1%) (Supplementary Table S3). Somewhat unexpectedly, the bacterial *rrn16*, *rrn23*, and *psbA* sequences also shared high nucleotide identities with entries in the virus database (Figure 5A). The Synechococcus *rrn23* was most closely related to the gene of stealth virus-1 (clone 3b43 T7) (*Cytomegalovirus*; Herpesviridae) (GenBank accession no. AF065756.1) [67], at 80% similarity (e-value < 1 × 10^−61^, 12% coverage). The *rrn16* and *rrn23* were most closely related to those of stealth virus (clone 3B43) (*Cytomegalovirus*; Herpesviridae) (GenBank accession no. AF191073.1) [68], at 80% similarity (e-value < 4 × 10^−163^, 54–56% coverage). The cyanobacterial *psbA* was most closely related to a gene found in the *Prochlorococcus* phage, P-TIM68 (Caudovirales, Myoviridae) (GenBank accession no. KM359505.1), at 78% similarity (e-value: 7 × 10^−120^, 66% coverage) and an uncultured cyanophage (GenBank accession no. HQ634189.1), at 77% similarity (e-value: 3 × 10^−148^; 87% coverage), the latter being extant and infectious in terrestrial mammals [68]. The amino acid sequences of *psaA*, *psaB*, *psbA*, and *psbD* of the three *Chlorellaceae* spp. also show close sequence similarities to those of viruses (Figure 5B).

**Figure 6:**
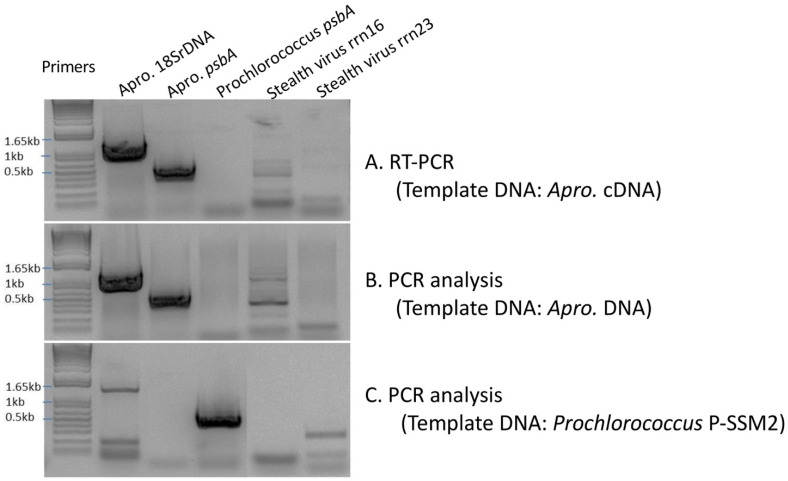
Results of the experiments designed to detect the presence of viral genes, *Prochlorococcus P-SSM2* photosystem II protein D1 (*psbA*), stealth virus 16S ribosomal RNA (*rrn16*), and 23S ribosomal RNA (*rrn23*). The cloned algal 18S rDNA was used as an internal control. (**A**) RT-PCR amplification was carried out using gene-specific primers for *Prochlorococcus* phage *psbA* sequence (588 bp), stealth virus *rrn16* (230 bp), and stealth virus *rrn23* (331 bp), using the gDNA as template. (**B**) The gDNA was subjected to conventional PCR amplification to detect the presence of viral genes. (**C**) The *Prochlorococcus* P-SSM2 lysate was subjected to conventional PCR amplification to detect the presence of *psbA*.

To determine whether the virus-like genes in *A. protothecoides* were viable, e.g., expressed, the viral gene and putative corresponding transcript were targeted by both PCR and reverse transcriptase (RT)-PCR using a primer pair that specifically amplifies *Prochlorococcus* phage *psbA*-like gene sequences (GenBank accession no. NC006883) and a primer pair for two stealth virus-like *rrn16* and *rrn23* sequences (GenBank accession no. AF191073). The results of the RT-PCR amplification from total genomic DNA (gDNA) using primers specific to the *A. protothecoides* 18S rRNA gene (18S rDNA), *Prochlorococcus* phage *psbA*, and stealth virus *rrn16/rrn23* indicated the absence of virus-like transcripts (Figure 6A and Supplementary Figure S3) and of PCR-amplifiable sequences from gDNA purified from *A. protothecoides* monocultures (Figure 6B and Supplementary Figure S3). We confirmed that the purified DNA from the *Prochlorococcus* culture was of high quality based on the presence of a robust band of high molecular weight of the *Prochlorococcus psbA* (Figure 6C and Supplementary Figure S3). Based on the results of the PCR and RT-PCR analyses, there were no detectable *Prochlorococcus* P-SSM2 phage-like transcripts expressed in the *A. protothecoides* suspension monoculture, suggesting that pro-phage and/or phage-like sequences recognizable in the chloroplast genome were not expressed in the algal cultures examined here. 

## 4. Discussion

The results of resequencing and comparative analysis of the chloroplast genome of *A. protothecoides* UTEX 25 inform us of the diverse gene composition and architecture of the algal chloroplast genome. Comparative analysis with its closest photosynthetic relatives, *C. variabilis* and *C. vulgaris,* provides new relevant clues about the photosynthetic capabilities of Chlorella and Auxenochlorella strains for current biofuel production. 

### 4.1. Genome Comparison 

Although the three Chlorellaceae studied here were similar in size, at 2–10 μm in diameter, the chloroplast genome of *A. protothecoides* was smaller (84 kbp) and more compact than those of *C. variabilis* and *C. vulgaris,* at 124 kbp and 150 kbp, respectively. Additionally, the latter two algal species harbor a higher abundance of non-coding sequence regions compared to *A. protothecoides*, of which the *A. protothecoides* chloroplast genome contained 19% non-coding sequence-relative content, compared to *C. variabilis* and *C. vulgaris*, at 46% and 53%, respectively. 

Global comparative analyses revealed that approximately 95% of genes of *A. protothecoides* were conserved in two other Chlorella species, except for unique genes *trnL-UAA*, *rps12_5*, and *Tils*, which were found only in *A. protothecoides*. Additionally, a gene encoding ChlI was uniquely duplicated in *A. protothecoides* cpDNA, whereas the predicted cell-cycle gene, *ycf62*, and *minE* involved in cell division were lacking in the *A. protothecoides* cpDNA. 

Phylogenetic analysis of 37 Trebouxiophyceae chloroplast genomes was consistent with previous analyses that placed *A. protothecoides* and *P. cutis* in the same clade (Figure 2). Our phylogenetic analysis was consistent with a study by Suzuki et al., which shows the closest species of *A. protothecoides* is heterotrophic green algae *P. cutis*, which lacks many photosynthetic-related genes [69]. 

In plants, RNA polymerases are multisubunit proteins comprising *rpoA, rpoB, rpoC1*, and *rpoC2* [70]. Accordingly, the genes *rpoA*, *rpoB*, *rpoC1*, and *rpoC2*, encoding RNA polymerase subunits, were identified in the three Chlorellaceae chloroplast genomes. 

To synthesize chlorophyll in a light-dependent context, three genes, are essential: *chlI*, *chlD*, and *chlH*. The results revealed that only the *chlI* is present in the three Chlorellaceae chloroplast genomes; therefore, the absence of *chlD* and *chlH* in the chloroplast genomes suggests that these genes are encoded in the nuclear genome. A search of the annotated genome sequence for *chlD* verified the presence of coding regions in the *A. protothecoides* nuclear genome; however, *chlH* was not present in the nuclear or chloroplast DNA [71]. By comparison, *chlD* and *chlH* were evident and annotated as hypothetical proteins in the nuclear genome of *C. variabilis* [61], although *C. vulgaris* encoded no detectable *chlI* and *chlH*. However, *chlD* and *chlH* have been identified in the nuclear genome of many higher plants [60,72,73]. 

### 4.2. Evolutionary Implications 

One possible explanation for the evolution of the relatively smaller cpDNA of *A. protothecoides* could be that the smaller size has aided its adaptation to new niches or specific environments. Such a scenario might be expected to result from the prospective evolutionary benefit of evolving a more streamlined, more efficient chloroplast genome particularly capable of responding to environmental stresses by lowering the energy required for multiplication and cell division, presumably associated with the smaller genome size. Additionally, the smaller-organelle genomes could be attributed to a ‘non-adaptive’ process mediated by TEs in gene duplication and/or in deletion of genomic contents [28,74,75], resulting in the expansion and contraction of non-coding regions in organelle genomes [76]. cpDNA analysis revealed that the three Chlorella species cpDNAs are without detectable TEs. However, a greater number of genetic mobile elements of group I and II introns were identified in the *C. variabilis* and *C. vulgaris* chloroplast genomes, suggesting that these distinct gene rearrangements, as well as the relative reduction in non-coding sequences compared to the *A. protothecoides* cpDNA, may be related to their presence. 

The cyanobacterial genes *rrn16* and *rrn23*, which showed sequence similarities to those in the cpDNA of *A. protothecoides,* were also identified in several cyanophages and stealth virus clones in mammals (Figure 5A). Mammalian stealth viruses belong to the family *Herpesviridae* and are known to have ancient aquatic origins and to have coevolved with marine cyanobacteria, as well as marine animals [77]. Consequently, marine cyanobacteria infected with stealth virus could have served as the source and means of horizontal gene transfer, mobilizing the virus-like genes into the green algae. These predicted homologs were identified in a marine cyanophage as functional cyanobacterial genes encoding the extant photosystem subunit I/II (PSI/II) [78]. Perhaps by utilizing cyanobacterial host genes, cyanophages have evolved increased fitness while also potentially enhancing their host’s fitness and survival [78]. Similarly, horizontal gene transfer can confer additional genomic variability to both the bacterium and the bacterial host. For example, horizontal gene transfer between bacteriophages and *Pseudomonas aeruginosa* has been shown to contribute genetic diversity linked to selective benefits considered responsible for the adaptation of the bacterium to its specific habitats [79]. 

The results of PCR and RT-PCR amplification to detect the gene and transcripts, respectively, corresponding to the marine virus-like sequences in *A. protothecoides* UTEX 25 culture indicate that the predicted virus-like genes, or portions of them, were present in the genome. However, transcripts expected to be detected if expressed were not amplified by RT-PCR for any of the predicted genes (Figure 6). The inability to detect predicted virus-like elements supports the hypothesis that marine viral signatures in the genome are the result of previous HGT events that may have been silenced by the host. Even so, based on the nature of the predicted virus-like gene functions, the acquisition of these genes/functions appear to be of great importance for these algae to thrive in marine habitats, given their predicted role in photosynthesis and protein synthesis, respectively. Conversely, several marine viruses encode a non-virus-oriented gene repertoire of certain amino acid biosynthetic pathways that are assumed to have been acquired from the host [80]. Additionally, certain *Chlorella*-infecting viruses are known to have evolved increased fitness by incorporating host genes through HGT. About 4–7% of all *chlorovirus* genomes are of bacterial origin, whereas 1–2% appear to have originated from plants [81]. For example, two proteins, elongation factor EF-3 (CL0450) and CL0511, which are encoded by a virus, NC64A, that infects *C. variabilis*, have recognizably shared sequence identities with *C. variabilis* homologs [81]. In addition, genes required for chitin metabolism in *C. variabilis* NC64A were found to be phylogenetically-related to the putatively homologous genes of the *Paramecium bursaria* Chlorella virus 1 [61], making it likely that these genes have been shuttled between *C. variabilis* and certain *Chlorella*-infecting viruses by HGT. Overall, evolutionarily, such inter-organismal genomic interplay is expected to contribute to algal cpDNA diversification. 

### 4.3. Future Biotechnological Impact

Despite the ability of *A. protothecoides* to produce abundant triacylglycerols (TAGs), its utilization for commercial biofuels has been impeded in part because of limited knowledge about fatty acid biosynthesis and TAG assembly, which are carried out in the endoplasmic reticulum (ER) [82]. The ability to transform the chloroplast genome would therefore be advantageous not only for TAG production but also for the production of various bioactive molecules (e.g., carotenoids, carbohydrates, proteins, etc.), specifically if it were possible to enhance heterologous protein synthesis with a site-specific transgene insertion instead of by using random genomic integration to minimize unintended phenotypic effects. However, the use of Chlorella for recombinant protein expression has not been practical because genetic tools required for stable transformation have been lacking [83]. Thus, the confirmation of the cpDNA sequence analyses reported herein is expected to inform chloroplast transformation aimed at elucidating lipid biosynthesis pathways in the chloroplast in order to gain understanding of downstream modulation of fatty acid synthesis and/or to increase expression of other useful triacylglycerides or tri-terpenes (hydrocarbons) from non-homologous algal species. Our genome assembly will help guide further modifications through better understanding of the architecture of these genes in multiple species. Understanding and comparison of genetic architecture can provide insight into the underlying mechanisms of different biofuel-producing phenotypes. Follow-on studies that link possible biomass production or carbon flux with the chloroplast genotypes presented here could possibly lead to new insights for improving carbon flux towards a desired biomass or lipid production. 

Finally, in addition to protein-coding genes, several plastid regulatory sequences (e.g., plastid-specific promoters, terminators, and 5′UTRs) can be mined, cloned, and used to drive transgene expression and direct homologous recombination through chloroplast transformation to affect gene knockouts and/or facilitate protein overproduction. Many chloroplast genes are involved in fatty acid production pathways, and their expression is regulated significantly under increased fatty acid production conditions, as shown in proteomics-based expression studies in other green algae [84]. The chloroplast genes and regulatory elements therefore provide excellent targets for fatty acid overproduction and regulation of carbon flux. 

## 5. Conclusions

Genomic comparative analyses of *A. protothecoides* with its two closest relatives, *C. variabilis* and *C. vulgaris*, indicated that many conserved genes between these three species are organized in colinear blocks. However, ample genomic rearrangements are also evident. Additionally, the cpDNA of *A. protothecoides* was smaller and more compact than that of *C. variabilis* and *C. vulgaris*, a scenario that is possibly due to fewer non-coding regions, which may be explained by the observed rearrangements. Smaller cpDNAs may confer evolutionary benefits to certain algal species, for example, increased fitness. Auxenochlorella and the two Chlorella strains used in this study harbor similar gene composition for photosystem subunit I/II and chlorophyll synthesis. This similarity may provide relevant new insights into the photosynthetic capabilities of Chlorella and Auxenochlorella strains for current biofuel production. Having strains that are adapted for efficient photosynthesis and growth and understanding the evolutionary adaptations involved can provide increased biomass yields and fatty acid biosynthesis for biofuel production. 

The fully annotated cpDNA sequence of *A. protothecoides* provides immediate access to plastid-encoding genes and composition, facilitating detailed studies to better understand cell division, as well as chlorophyll biosynthetic, photosynthetic, and fatty acid biosynthetic processes. These provide excellent targets for engineering of such species for enhanced biofuel production. Finally, the comparative genomic analysis of three closely related Chlorellaceae species provides additional insights into chloroplast biology and evolutionary processes, which are important for further understanding the close species–species interactions and possible genetic transfer that occurs in complex environmental mixtures.

## Figures and Tables

**Table 1:** Results of the comparison with the diatom *Thalassiosira pseudonana*, genomic libraries that harbored the predicted transposable element (TE), and repeated sequences of the cpDNA summarized with respect to TE elements, small RNA, simple repeats, and low-complexity DNAs for the chloroplasts of the three algal species of interest. * Repeats that appeared to be fragmented by insertions or deletions were counted as one element.

	*A. protothecoides* (GenBank Accession No. KC631634.1)			*C. variabilis* (GenBank Accession No. KJ718922.1)			*C. vulgaris* (GenBank Accession No. NC001865.1)		
	Number *	Length (bp)	Percentage (%)	Number *	Length (bp)	Percentage (%)	Number *	Length (bp)	Percentage (%)
TE elements	-	-	-	-	-	-	-	-	-
Small RNA	3	1892	2.23	2	1533	1.23	3	936	0.62
Simple repeats	3	156	0.18	1	34	0.02	27	958	0.63
Low complexity	135	10,129	11.9	45	1858	1.49	168	7725	5.12

## Data Availability

All data generated or analyzed during this study are included in this published article and its Supplementary Materials.

## Supplementary Materials

The following supporting information can be downloaded at: https://www.mdpi.com/article/10.3390/life12030458/s1. Figure S1: Global chloroplast genome sequence alignment of *Auxenochlorella protothecoides*, *Chlorella variabilis*, and *Chlorella vulgaris*. Local co-linear blocks (LCB) were identified as regions of homologous sequences shared by all three species. The lines connecting the genomes indicate orthologous gene clusters found in all three chloroplast genomes. The peaks in the LCB block indicate a similarity profile within each gene cluster. Areas that are completely white were not aligned and probably contain sequence elements specific to a particular genome. The multiple chloroplast genome alignment was conducted using Mauve software. Figure S2: Phylogenetic relationships among the Trebouxiophyceae chloroplast genomes using Maximum Likelihood. Numbers placed at major nodes indicate bootstrap confidence values at ≥70% for 1000 iterations. Figure S3: An original gel image used in Figure 6. Reverse Transcriptase-Polymerase Chain Reaction (RT-PCR) on *Auxenochlorella protothecoides* cDNA for examination of presence of viral genes (Prochlorococcus P-SSM2 *psbA*, Stealth virus *rrn16* and *rrn23*). 18S ribosomal DNA is used as internal control. A. RT-PCR was performed using gene specific primers of *A. protothecoides psbA*, *Prochlorococcus* phage *psbA* (588nt), Stealth virus *rrn16* (230nt) and Stealth virus *rrn23* (331nt). B. Conventional PCR was performed on *C. protothecoides* gDNA to show the presence of viral genes. C. Conventional PCR was performed on *Prochlorococcus* P-SSNM2 lysate to confirm the presence of *psbA*. Table S1: Inventory of predicted genes within the *Auxenochlorella* *protothecoides*, *Chlorella variabilis*, and *Chlorella vulgaris* chloroplast genomes. trn: transfer RNA; rrn: ribosomal RNA; rpl: ribosomal protein large subunit; rrs: ribosomal protein small subunit; chl: chlorophyll biosynthesis, psa/psb: photosystem I/II subunits; rpo: RNA polymerase subunit; ycf: hypothetical chloroplast ORF; cyst: sulfate transport ATP-binding protein; ftsH/minE: plastid division; cemA: envelope membrane. †Gene including intron. *Not found in the other Chlorellaceae species in Table 1. § Partial sequences. The copy number for each is indicated parenthetically. Table S2: Protein sequences (*n* = 26) identified based on the best-fitting partitioning (*n* = 17) and protein substitution models. The best-fitting partition and protein substitution scheme for 26 proteins, resulting in 17 partitions, determined by PartitionFinder version 2.1.1. Table S3: Results of BLASTn searches for the *Auxenochlorella protothecoides* UTEX 25 cpDNA against genome sequences of the Cyanobacteria (taxid:1117) available in the National Center for Biotechnology Information (NCBI) GenBank database. For top hit selection, the following filtering criteria (percent identity: >86%, E-value: 0, and query coverage: >1%) were used.
