# Supplementary material for: Comparison of Auxenochlorella protothecoides and Chlorella spp. Chloroplast Genomes: Evidence for Endosymbiosis and Horizontal Virus-like Gene Transfer
Source: Life (Basel). 2022 Mar 20;12(3):458. doi: 10.3390/life12030458 (PMC8955559; doi:10.3390/life12030458)
Supplement: Supplementary file 1 [file life-12-00458-s001.zip › life-1613100-supplementary.pdf]

life

Article

Comparison of *Auxenochlorella protothecoides* and *Chlorella* spp. Chloroplast Genomes: Evidence for Endosymbiosis and Horizontal Virus-like Gene Transfer

Sang-Hyuck Park [1,2], John A. Kyndt [3,*] and Judith K. Brown [1]

[1] School of Plant Sciences, The University of Arizona, Tucson, AZ 85721, USA; sanghyuck.park@csupueblo.edu (S.-H.P.); jbrown@ag.arizona.edu (J.K.B.)
[2] Institute of Cannabis Research, Colorado State University-Pueblo, Pueblo, CO 81001, USA
[3] College of Science and Technology, Bellevue University, Bellevue, NE 68005, USA
* Correspondence: jkyndt@bellevue.edu

Supplementary Figures

Citation: Park, S.-H.; Kyndt, J.A.; Brown, J.K. Comparison of *Auxenochlorella protothecoides* and *Chlorella* spp. Chloroplast Genomes: Evidence for Endosymbiosis and Horizontal Virus-like Gene Transfer. *Life* **2022**, *12*, 458. https://doi.org/10.3390/life12030458

Academic Editor: Francois Lefort

Received: 10 February 2022
Accepted: 16 March 2022
Published: 20 March 2022

Publisher's Note: MDPI stays neutral with regard to jurisdictional claims in published maps and institutional affiliations.

Copyright: © 2022 by the author. Licensee MDPI, Basel, Switzerland. This article is an open access article distributed under the terms and conditions of the Creative Commons Attribution (CC BY) license (https://creativecommons.org/licenses/by/4.0/).

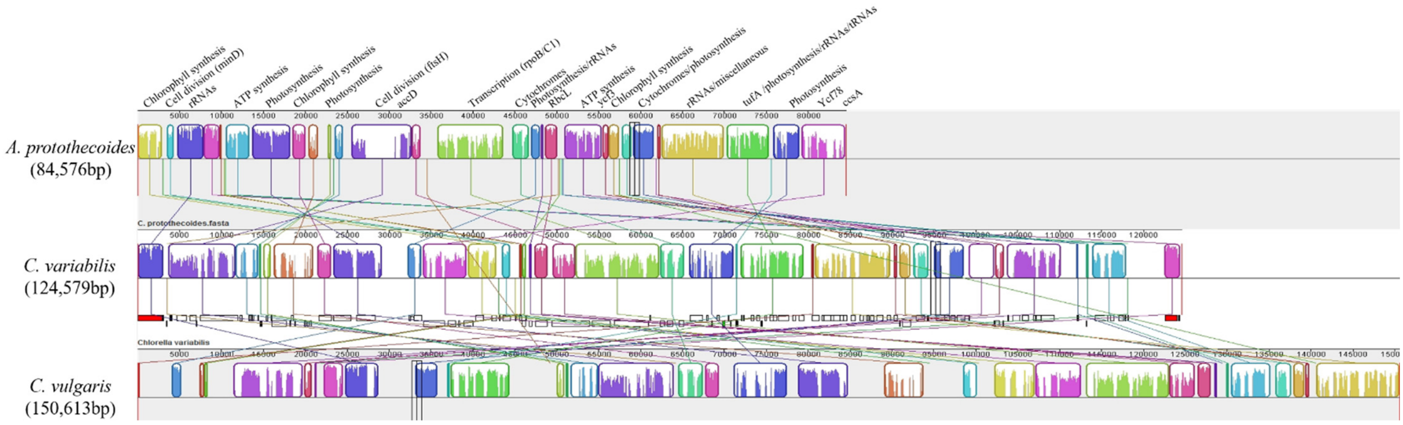

Figure S1. Global chloroplast genome sequence alignment of *Auxenochlorella protothecoides*, *Chlorella variabilis*, and *Chlorella vulgaris*. Local co-linear blocks (LCB) were identified as regions of homologous sequences shared by all three species. The lines connecting the genomes indicate orthologous gene clusters found in all three chloroplast genomes. The peaks in the LCB block indicate a similarity profile within each gene cluster. Areas that are completely white were not aligned and probably contain sequence elements specific to a particular genome. The multiple chloroplast genome alignment was conducted using Mauve software (http://darlinglab.org/mauve/user-guide/introduction.html).

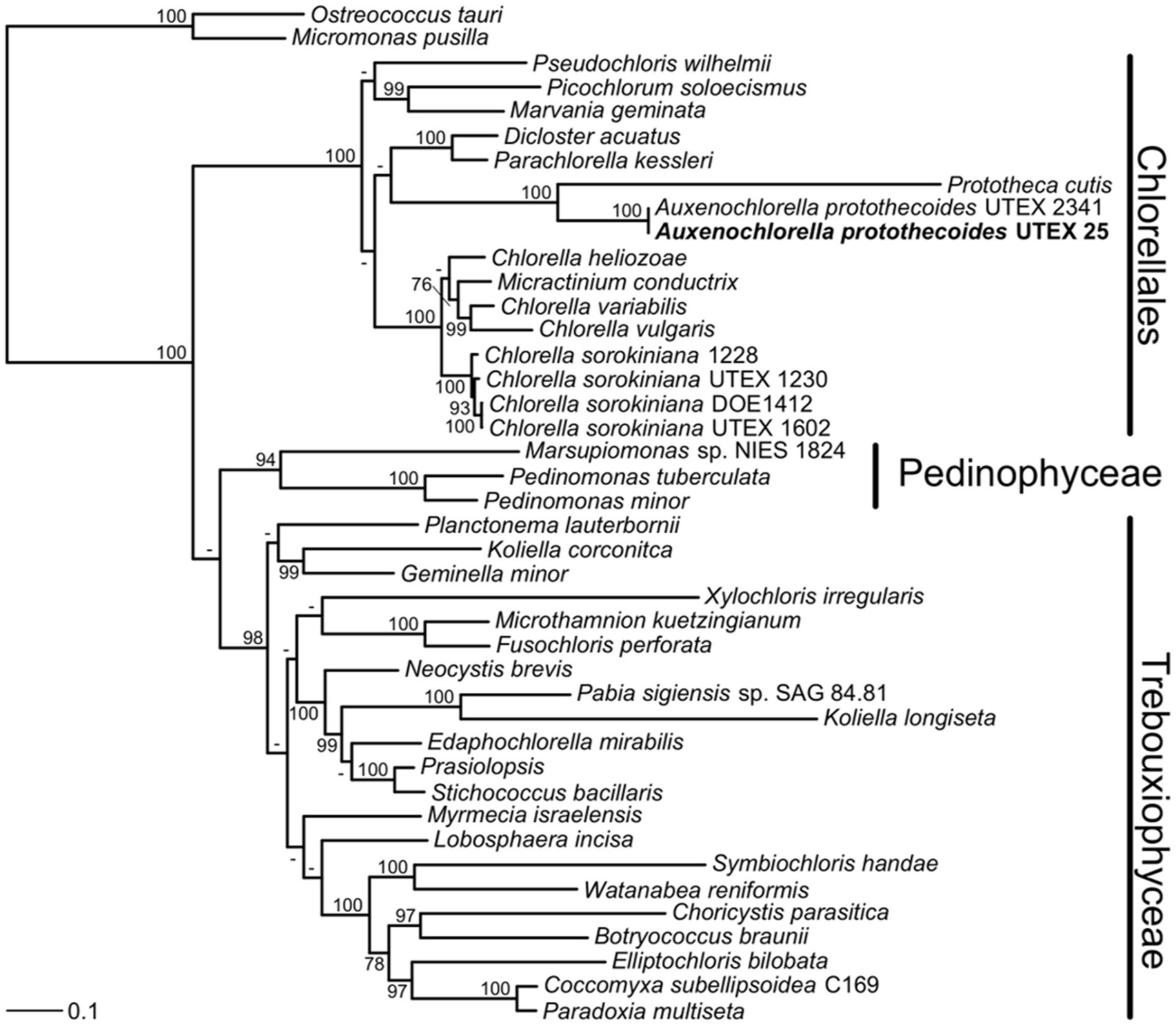

Figure S2. Phylogenetic relationships among the Trebouxiophyceae chloroplast genomes using Maximum Likelihood. Numbers placed at major nodes indicate bootstrap confidence values at ≥ 70% for 1000 iterations.

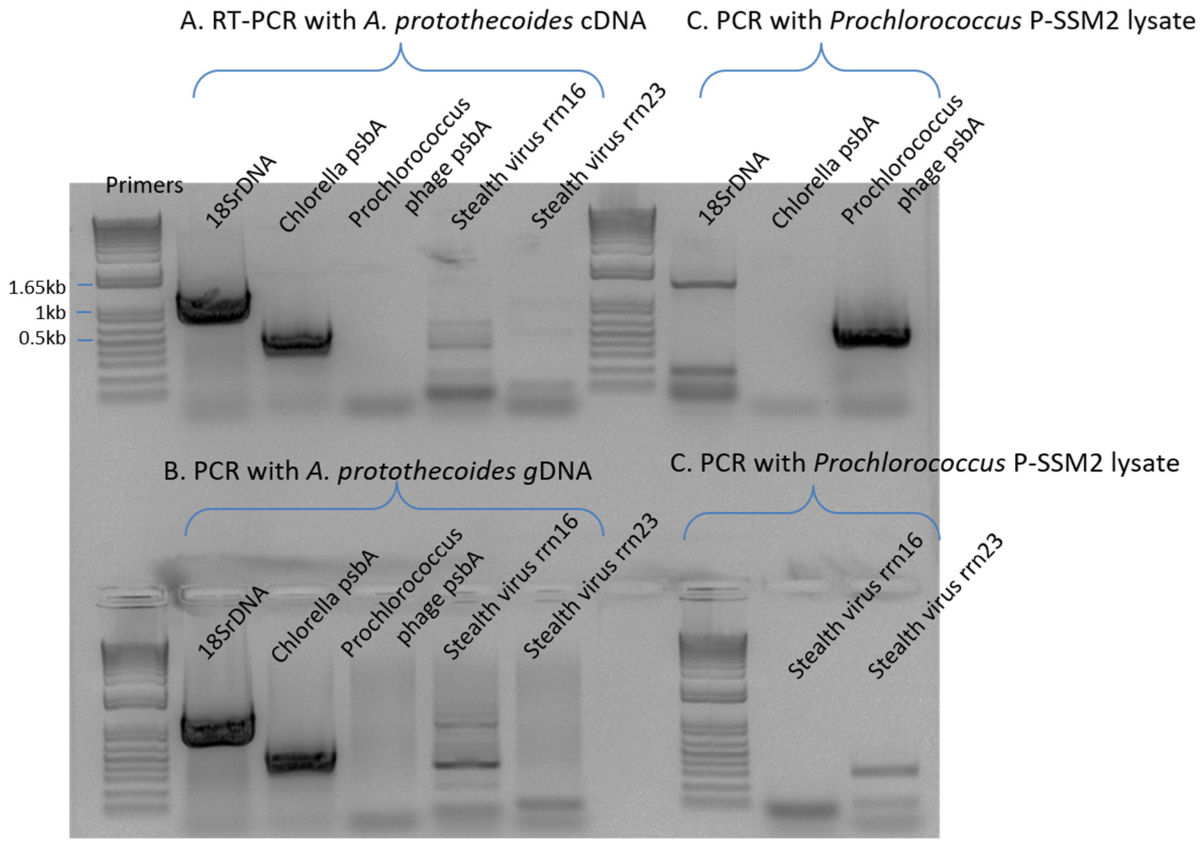

Figure S3. An original gel image used in Figure 6. Reverse Transcriptase-Polymerase Chain Reaction (RT-PCR) on *Auxenochlorella protothecoides* cDNA for examination of presence of viral genes (Prochlorococcus P-SSM2 *psbA*, Stealth virus *rrn16* and *rrn23*). 18S ribosomal DNA is used as internal control. A. RT-PCR was performed using gene specific primers of *A. protothecoides psbA*, *Prochlorococcus* phage *psbA* (588nt), Stealth virus *rrn16* (230nt) and Stealth virus *rrn23* (331nt). B. Conventional PCR was performed on *C. protothecoides* gDNA to show the presence of viral genes. C. Conventional PCR was performed on *Prochlorococcus* P-SSNM2 lysate to confirm the presence of *psbA*.

Supplementary Tables

Table S1. Inventory of predicted genes within the *Auxenochlorella protothecoides*, *Chlorella variabilis*, and *Chlorella vulgaris* chloroplast genomes. trn: transfer RNA; rrn: ribosomal RNA; rpl: ribosomal protein large subunit; rrs: ribosomal protein small subunit; chl: chlorophyll biosynthesis, psa/psb: photosystem I/II subunits; rpo: RNA polymerase subunit; ycf: hypothetical chloroplast ORF; cysA: sulfate transport ATP-binding protein; ftsH/minE: plastid division; cemA: envelope membrane. †Gene including intron. *Not found in the other Chlorellaceae species in Table 1. § Partial sequences. The copy number for each is indicated parenthetically.

A. protothecoides **UTEX 25 84,576 bp (114 genes) (GC: 30.8%)**	***C. variabilis*** **124,579 bp (113 genes) (GC: 34%)**	***C. vulgaris*** **150,613 bp (120 genes) (GC: 32%)**
**Photosynthesis**		
**Photosystem I**		
psaA psaB psaC psaI psaJ psaM	*psaA psaB psaC psaI psaJ psaM*	*psaA psaB psaC psaI psaJ psaM*
**Photosystem II**		
psbA psbB psbC psbD psbE psbF psbH psbI psbJ psbK psbL psbM psbN psbT psbZ	*psbA (2) psbC(3) psbD psbE psbF psbH psbI psbJ psbK psbL psbM psbN psbT psbZ psi_psbT*	*psbA psbC psbD psbE psbF psbH psbI psbJ psbK psbL psbM psbN psbT psbZ psi_psbT*
**Cytochrome**		
petA petB petD petG petL	*petA petB petD petG petL*	*petA petB petD petG petL*
**ATP synthesis**		
atpA atpB atpE atpF atpH atpI	*atpA atpB atpE atpF atpH atpI*	*atpA atpB atpE atpF atpH atpI*
**Chlorophyll synthesis**		
chlB chlI (2) chlL chlN	*chlB chlI chlL †chlN*	*chlB chlI †chlL chlN*
**Rubisco**		
rbcL	*rbcL*	*rbcL*
**Transcription/translation**		
RNA polymerase		
rpoA rpoB rpoC1 rpoC2	*rpoA rpoB rpoC1 rpoC2*	*rpoA rpoB rpoC1 rpoC2, §rpoC2*
**Translation factor**		
infA tufa	*infA tufA*	*infA tufA*
**Chloroplast division**		
minD ftsH	*minD ftsH*	*minD *minE ftsH*
**Ribosomal RNAs**		
rrn5 rrn16 rrn23 (2)	*rrn5 rrn16 rrn23(2)*	*rrn5 rrn16 (4) rrn23*
**Ribosomal proteins**		
**Large subunits**		
rpl2 rpl5 rpl12 rpl14 rpl16 rpl19 rpl20 rpl23 rpl32 rpl36	*rpl2 rpl5 rpl12 rpl14 rpl16 rpl19 rpl20 rpl23 rpl32 rpl36*	*rpl2 rpl5 rpl12 rpl14 rpl16 rpl19 rpl20 rpl23 rpl32 rpl36*
**Small subunits**		
rps2 rps3 rps4 rps7 rps8 rps9 rps11 rps12 rps12_5end rps14 rps18 rps19	*rps2 rps3 rps4 rps7 rps8 rps9 rps11 rps12 rps12_3end rps14 rps18 rps19*	*rps2 rps3 rps4 rps7 rps8 rps9 rps11 rps12 rps12_3end rps14 rps18 rps19*
**Transfer RNA**		
trnA-UGC trnC-GCA trnD-GUC trnE-UUC trnF-GAA trnfM-CAU	*trnA-UGC trnC-GCA(2) trnD-GUC trnE-UUC trnF-GAA trnfM-CAU trnG-*	*trnA-UGC trnC-GCA trnD-GUC trnE-UUC trnF-GAA trnfM-CAU trnG-GCC(2)*

trnG-GCC trnG-UCC trnH-GUG trnH-GUG trnI-CAU trnI-GAU trnK-UUU trnL-GAG trnL-UAA trnL-UAG trnM-CAU trnN-GUU trnP-GGG trnP-UGG trnQ-UUG trnR-ACG trnR-CCG trnR-UCU trnS-GCU trnS-GGA trnS-UGA trnT-GGU trnT-UGU trnV-UAC trnW-CCA trnY-GUA	*GCC(2) trnG-UCC trnH-GUG trnI-CAU trnI-GAU trnK-UUU trnL-CAA trnL-GAG trnL-UAG trnM-CAU trnN-GUU trnP-GGG trnP-UGG trnQ-UUG trnR-ACG trnR-CCG trnR-UCU trnS-GCU trnS-GGA trnS-UGA trnT-GGU trnT-UGU trnV-UAC trnW-CCA trnY-GUA*	*trnG-UCC trnH-GUG trnI-CAU trnI-GAU trnK-UUU trnL-CAA trnL-GAG trnL-UAG trnM-CAU trnN-GUU trnP-GGG trnP-UGG trnQ-UUG trnR-ACG trnR-CCG trnR-UCU trnS-GCU trnS-GGA trnS-UGA trnT-GGU trnT-UGU trnV-UAC trnV-UAC trnW-CCA trnY-GUA*
**Fatty acid biosynthesis**		
accD	*accD*	*accD*
**Others**		
ccsA cemA clpP cysT TilS ycf3 ycf4 ycf12 ycf78	*ccsA cemA clpP cysA cysT ycf3 ycf4 ycf12 ycf78*	*ccsA cemA(2) clpP cysA cysT ycf3 ycf4 ycf12 *ycf62 ycf78*

Table S2. Protein sequences (n = 26) identified based on the best-fitting partitioning (n = 17) and protein substitution models. The best-fitting partition and protein substitution scheme for 26 proteins, resulting in 17 partitions, determined by PartitionFinder version 2.1.1.

Partition number	Protein	Description	Number of sites	Substitution model
1	*atpB*	ATP synthase beta-subunit	598	LG4X
2	*psaA*	P700 chlorophyll a-apoprotein A1	1486	MTZOA+I+G+F
	psaB	P700 chlorophyll a-apoprotein A2		
3	*psaC*	subunit VII of photosystem I (Fe-Spolypeptide)	556	LG4X
	rbcL	large subunit of Rubisco		
4	*psbC*	photosystem II CP43 apoprotein	488	LG4X
5	*RPL12*	ribosomal protein L12	136	LG+G+F
6	*RPL14*	ribosomal protein L14	217	LG4X
	RPS19	ribosomal protein S19		
7	*RPL16*	ribosomal protein L16	144	LG4X
8	*RPS4*	ribosomal protein S4	654	LG+I+G+F
	RPS8	ribosomal protein S8		
	RPL19	ribosomal protein L19		
9	*RPL5*	ribosomal protein L5	494	LG4X
	RPL2	ribosomal protein L2		
10	*RPL20*	ribosomal protein L20	122	LG4X
11	*RPL23*	ribosomal protein L23	737	JTTDCMUT+G+F
	RPS18	ribosomal protein S18		
	RPS2	ribosomal protein S2		
	RPL32	ribosomal protein L32		
12	*RPS11*	ribosomal protein S11	136	LG4X
13	*RPS12*	ribosomal protein S12	127	CPREV+I+G
14	*RPS14*	ribosomal protein S14	102	LG4X
15	*RPS3*	ribosomal protein S3	425	MTREV+I+G+F
16	*RPS7*	ribosomal protein S7	157	LG4X
17	*RPS9*	ribosomal protein S9	205	CPREV+I+G

Table S3. Results of BLASTn searches for the *Auxenochlorella protothecoides* UTEX 25 cpDNA against genome sequences of the Cyanobacteria (taxid:1117) available in the National Center for Biotechnology Information (NCBI) GenBank database (https://blast.ncbi.nlm.nih.gov/Blast.cgi?PROGRAM=blastn&PAGE_TYPE=BlastSearch&LINK_LOC=blasthome). For top hit selection, the following filtering criteria (percent identity: >86%, E-value: 0, and query coverage: >1%) were used.

	NCBI GenBank Accession #	Cyanobacterium	Gene	Query Coverage	Percent Identity	Gaps
1	EU703214.1	Uncultured cyanobacterium	16S rRNA	1%	88.94%	54/1420(3%)
2	FJ901770.1	Uncultured cyanobacterium	16S rRNA	1%	89.49%	47/1313(3%)
3	CP060212.1	*Limnospira* sp. BM	23S rRNA	9%	86.55%	31/1323(2%)
4	MT863733.1	*Limnospira* sp. BM	23S rRNA	2%	86.48%	32/1324(2%)
5	AP012549.1	Cyanobacterium endosymbiont of *Epithemia turgida* isolate EtSB Lake Yunoko	23S rRNA	4%	87.98%	31/1190(2%)
6	NR076532.1	*Crocosphaera subtropica* ATCC 51142	23S rRNA	2%	87.64%	29/1189(2%)
7	CP000806.1	*Crocosphaera subtropica* ATCC 51142	23S rRNA	4%	87.64%	29/1189(2%)
8	AP018202.2	*Synechococcus vulcanus* NIES-2134	23S rRNA	4%	87.53%	29/1187(2%)
9	AP018341.1	Cyanobacterium endosymbiont of *Rhopalodia gibberula*	23S rRNA	4%	87.39%	31/1190(2%)
10	AY584507.1	*Euhalothece* sp. BAA001	23S rRNA	2%	86.29%	28/1240(2%)
11	NR102529.1	*Stanieria cyanosphaera* PCC 7437	23S rRNA	2%	86.33%	24/1222(1%)
12	CP003653.1	*Stanieria cyanosphaera* PCC 7437	23S rRNA	6%	86.33%	24/1222(1%)
13	CP051167.1	*Oxynema* sp. AP17	23S rRNA	4%	86.81%	16/1183(1%)
14	NR076263.1	*Synechococcus* elongatus PCC 6301	23S rRNA	2%	86.75%	20/1185(1%)
15	CP000100.1	*Synechococcus* elongatus PCC 7942 = FACHB-805	23S rRNA	4%	86.75%	20/1185(1%)
16	LC455673.1	Kamptonema laetevirens NIES-31	23S rRNA	2%	86.51%	25/1201(2%)
17	X00512.1	*Synechococcus* elongatus PCC 6301	23S rRNA	2%	86.67%	22/1185(1%)
18	AP017375.1	*Stanieria* sp. NIES-3757	23S rRNA	8%	86.59%	19/1186(1%)
19	CP051168.1	*Phormidium* sp. ETS-05	23S rRNA	4%	86.59%	21/1186(1%)
20	CP000240.1	*Synechococcus* sp. JA-2-3B'a(2-13)	23S rRNA	4%	86.62%	26/1188(2%)
21	LC455660.1	*Planktothrix agardhii*	23S rRNA	2%	86.46%	20/1189(1%)
22	LC455656.1	*Planktothrix agardhii*	23S rRNA	2%	86.46%	20/1189(1%)
23	AP017991.1	*Planktothrix agardhii* NIES-204	23S rRNA	5%	86.46%	20/1189(1%)
24	LO018304.1	*Planktothrix agardhii*	23S rRNA	5%	86.46%	20/1189(1%)
25	CP000239.1	*Synechococcus* sp. JA-3-3Ab	23S rRNA	4%	86.48%	26/1191(2%)
26	NR076758.1	*Nostoc azollae* 0708	23S rRNA	2%	86.45%	23/1188(1%)
27	CP002059.1	*Nostoc azollae* 0708	23S rRNA	4%	86.45%	23/1188(1%)
28	AP018254.1	*Calothrix* sp. NIES-3974	23S rRNA	6%	86.29%	20/1189(1%)
29	LC455612.1	*Phormidium* sp. NIES-2121	23S rRNA	2%	86.18%	19/1187(1%)
30	CP016483.1	*Synechococcus* sp. PCC 8807	23S rRNA	4%	86.16%	20/1185(1%)
31	CP040360.1	*Synechococcus* sp. PCC 11901	23S rRNA	4%	86.08%	20/1185(1%)
32	CP013998.1	*Synechococcus* sp. PCC 73109	23S rRNA	4%	86.08%	20/1185(1%)
33	CP000951.1	*Synechococcus* sp. PCC 7002	23S rRNA	4%	86.08%	20/1185(1%)
34	NR102540.1	Gloeocapsa sp. PCC 7428	23S rRNA	2%	86.03%	22/1188(1%)
35	NR102530.1	*Cylindrospermum stagnale* PCC 7417	23S rRNA	2%	86.04%	23/1189(1%)
36	CP003646.1	Gloeocapsa sp. PCC 7428	23S rRNA	7%	86.03%	22/1188(1%)
37	CP003642.1	*Cylindrospermum stagnale* PCC 7417	23S rRNA	4%	86.04%	23/1189(1%)
